# Ten-Year Trend Analysis of Mortality Due to External Causes of Injury in People with Disabilities, South Korea, 2008–2017

**DOI:** 10.3390/ijerph18073672

**Published:** 2021-04-01

**Authors:** Ye-Soon Kim, Sooyoung Kwon, Seung Hee Ho

**Affiliations:** Department of Healthcare and Public Health Research, Rehabilitation Research Institute, Korea National Rehabilitation Center, Seoul 01022, Korea; yesoon@korea.kr (Y.-S.K.); skwon2019@korea.kr (S.K.)

**Keywords:** people with disability, mortality, injury, external causes of injury

## Abstract

External causes of injury are major contributors to mortality among people with disabilities. We analyzed the 10-year trend (2008–2017) of mortality attributed to external causes of injury among people with disabilities. We conducted an observational, population-based, retrospective, cross-sectional study among people with disabilities in South Korea. The database was compiled by merging two data sets: registered people with disabilities during 2008–2017 from the Ministry of Health and Welfare, and the data published by the Korea National Statistical Office. Between 2008 and 2017, the all-cause mortality among people with disabilities showed a rising trend and increased from 2641 per 100,000 in 2008 to 2751 per 100,000 in 2017. During this 10-year period, 6.5–9.2% of the total number of deaths were caused by injuries. Disabilities that were associated with a high crude mortality rate shared the same three most frequent causes of death: suicide, motor vehicle crashes, and falling. Mortality due to external causes of injury increased among older people with disabilities. Thus, effective strategies are required to decrease preventable deaths caused by unintentional injuries among people with disabilities.

## 1. Introduction

Injury is one of the major causes of death in any population. Injuries pose a significant health burden in all populations, regardless of age, sex, income, or geographical region [1,2]. Globally, almost 5.8 million people die from injuries every year. This accounts for 10% of all deaths worldwide [3,4].

Unintentional injury is any injury that is not caused on purpose or with intention to harm. Since not all unintentional injuries are random events, and some can be prevented, it is usually not appropriate to use the word “accident” to define an unintentional injury. Unintentional injuries can be further classified according to external causes such as motor vehicle crashes, falls, poisoning, drowning, burns, etc. [5,6]. In Korea, injury is a significant medical and public health concern. In 2017, among the total deaths in Korea, the number of fatalities due to external causes of injury (ECIs) was 27,154 (9.5%), and the ECI-specific mortality rate was 53.0 per 100,000 people. The most frequent causes of ECI-related mortality were suicide (24.3%), motor vehicle crashes (9.8%), and falls (5.2%) [7,8]. These data are published annually by the Korea National Statistical Office.

In 2015, the Korean government put forth an act to guarantee the right to health and access to medical services for people with disabilities, which was implemented in 2017 [9]. Article 11 of the act specifies the maintenance of health and welfare statistics for people with disabilities. However, the maintenance and documentation of such statistics, including statistics related to specific causes of death among people with disabilities, are still insufficient [10]. Furthermore, in previous studies, the study populations were often limited to specific disability groups or were based on small sample sizes [11,12,13]. It is well established that people with disabilities have worse health and reduced survival rates than the general population; however, empirical data regarding this have not been well characterized [14,15]. Hence, in this study, we aimed to examine ECI-specific mortality trends in people with disabilities in South Korea from 2008 to 2017.

## 2. Materials and Methods

### 2.1. Data Sources

As of the end of the analysis period (2008–2017), the database evaluated in this study was established by merging data on people with disabilities registered at the Ministry of Health and Welfare and data on cause of mortality recorded by the Korea National Statistical Office [16]. The disability registration data included variables such as sex, age, major disability type, and comprehensive disability grade. If none of these variables existed, they were treated as missing and excluded from the analysis. Mortality according to ECIs for people with disabilities was classified according to the death certificate data, International Disease Classification (ICD-10) codes, and the World Health Organization (WHO)’s 103 classification criteria [17]. For this analysis, we focused on the main causes of death because of ECI, which are published annually by the Korea National Statistical Office. These causes of deaths can be categorized as follows: (1) motor vehicle crashes (ICD-10 codes V01–V99), (2) falls (ICD-10 codes W00–W19), (3) drowning (ICD-10 codes W65–W74), (4) burns (ICD-10 codes X00–X09), (5) poisoning (ICD-10 codes X40–X49), (6) suicide (ICD-10 codes X60–X85), and (7) homicide (ICD-10 codes X85–Y09). There were also miscellaneous ECI categories comprising deaths in categories (1) to (7) that were not included in the analysis, such as other accidental threats to breathing (ICD-10 codes W75–W84), accidental exposure to other or unspecified factors (ICD-10 codes X58–X59), events of undetermined intent (ICD-10 codes Y10–Y34), and sequelae of external causes of morbidity and mortality (ICD-10 codes Y85–Y89).

This study was approved by the National Rehabilitation Institute Clinical Research Review Committee (NRC-2012-04-026).

### 2.2. Registered People with Disabilities

People with disabilities are defined as persons registered as disabled with the Korean government. According to the Korean Disabled Persons Welfare Act, there is a registration and grading system for people with disabilities [16]. The law categorizes people with disabilities into the following 15 types: physical disability, disability with a brain lesion, visual impairment, hearing impairment, speech impairment, intellectual disability, autistic disorder, mental disorder, kidney dysfunction, cardiac dysfunction, respiratory dysfunction, liver dysfunction, facial dysfunction, intestinal fistula or urinary fistula, and epilepsy. This law has specific criteria for determining disability. The ratings for each type of disability range from 1 to 6. Grade 1 refers to the most severe disability, while grade 6 refers to mild disability. Usually, grades 1 to 3 are said to be people with severe disabilities, and grade 4 to 6 are said to be people with mild disabilities. If there are multiple disabilities, both the main disability and a comprehensive disability grade are given.

### 2.3. Statistical Analysis

We presented descriptive statistics for all-cause mortality and mortality due to ECIs among people with disabilities compared with that of the general population in South Korea. The mortality rate was calculated from the crude and the age-adjusted mortality rates. The crude mortality rate is the ratio of the number of deaths during the year to the average population in that year. The value is expressed per 100,000 inhabitants. The age-adjusted mortality rate is the ratio of the number of deaths in a given age group to the population in that age group expressed per 100,000 population. Data were analyzed using SAS 9.4.

## 3. Results

The crude all-cause mortality rates among people with disabilities ranged from 2400 to 2800 per 100,000 people in the 10-year period, which was about five times higher than that of the general population. The ECI-specific crude mortality rate of people with disabilities ranged from 6.5% to 9.2% in 10 years, whereas that of the general population was from 9.5% to 12.8%. People with disabilities had lower ECI-specific crude mortality rates than the general population, and the rate of ECI-related deaths among people with disabilities and the general population showed a decreasing trend (Table 1). The crude ECI-specific mortality rate of people with disabilities decreased from 237.7 per 100,000 population to 178.4 per 100,000 between 2008 and 2017, representing a decrease of 24.9% over the course of 10 years. The crude ECI-specific mortality rate of the general population decreased from 61.7 per 100,000 in 2008 to 53.0 per 100,000 in 2017, representing a decrease of 14.1% over the 10-year period.

**Figure 1:**
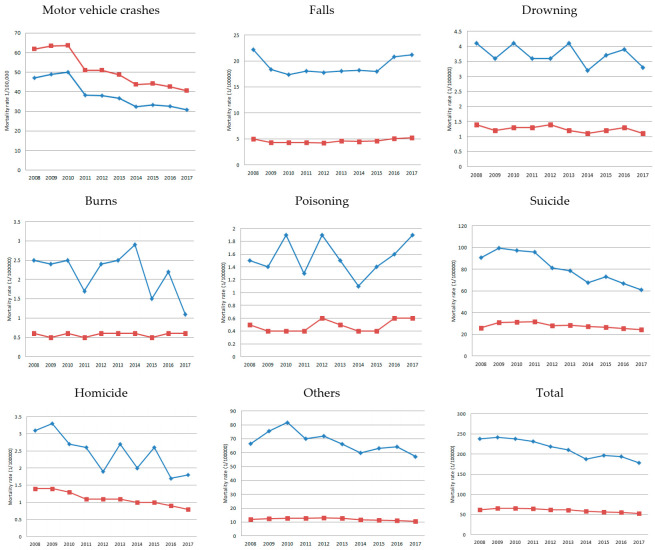
Trends of mortality rate due to external causes of injury (ECIs) (per 100,000) in South Korea, 2008–2017. The blue line is people with disabilities, and the red line is general populations.

The crude mortality rate due to ECIs of people with disabilities was approximately three times higher than that of the general population. Suicide, motor vehicle crashes, and falls were the leading causes of death among people with disabilities and the general population. For the general population, mortality due to ECIs gradually decreased in the 10-year period. Regarding people with disabilities, deaths due to motor vehicle crashes, falls, drowning, burns, and suicide were lower in 2017 than in 2008, and deaths due to addictions were slightly higher in 2017 than in 2008. Regarding the general population, deaths due to motor vehicle crashes, drowning, and suicide reduced from 2008 to 2017. On the other hand, deaths due to falls increased, and burns and poisoning had similar mortality rates during the 10-year period (Figure 1).

Both men and women with disabilities tended to have higher ECI-specific mortality rates with increasing age. Men were approximately twice as likely to experience an ECI than women. Suicide, motor vehicle crashes, and falls were the leading ECIs among men, and suicide, falls, and motor vehicle crashes were the most frequently experienced ECIs among women. The most frequent type of ECI experienced by teenagers was falls, and the highest suicide-related mortality rates were observed among people ≥20 years of age (Table 2).

In 2017, among people with disabilities, the ECI-specific crude mortality rates were the highest among those with a respiratory disorder, a mental disorder, and epilepsy. For 12 types of disability, the most prevalent cause of mortality was suicide, though this was not the case for those with an intellectual disorder, autism, and heart disorders. As a result of calculating the age-adjusted ECI-specific crude mortality rate, the ECI-specific standardized mortality rate was the highest among people with mental disorders, respiratory disorders, and liver disorders. Suicide was the leading cause of mortality among those with mental disorders. People with kidney failure had the highest standardized mortality rate due to motor vehicle crashes and falls (Table 3).

In all the age categories, the most prevalent ECIs were suicide, motor vehicle crashes, and falls. Suicide was the most prevalent ECI among people in their 20s, and motor vehicle crashes were the most prevalent ECIs among people in their 80s (Table 4).

## 4. Discussion

In the present study, we found that the decrease in mortality rate due to ECIs of people with disabilities (24.9%) between 2008 and 2017 was greater than that in the general population (14.1%). These results were similar to those found in other studies [18,19,20,21,22]. The reduction in ECI-specific mortality may be attributed to major improvements in the Korean emergency medical system. In 1990, a department of emergency medicine was established at emergency centers that specialized in treatment of trauma patients; thereafter, the preventable ECI-specific mortality rate reduced [23]. However, suicide is a major public health issue worldwide because it is one of the leading causes of premature death. In particular, the incidence of suicide has increased rapidly since 1997 in Korea and has thus emerged as a serious health and social problem [24].

In this study, although the mortality rate due to suicide steadily decreased during the 10-year study period, suicide represented the most prevalent cause of ECI-specific mortality. Specifically, in 2017, the mortality rate due to suicide was 61.2 per 100,000 among people with disabilities, whereas that in the general population was 24.3 per 100,000. Hence, the mortality rate due to suicide among people with disabilities was about 2.5 times higher than that among the general population. According to previous studies, the risk factors of the high suicide rate included a history of prior psychiatric hospitalization, comorbid physical disabilities, loneliness, sadness, depression, or anxiety in people with intellectual disability [25,26].

Regarding the results stratified by type of disability, the all-cause mortality rate among ECI was highest among those with respiratory disorders (204.5 per 100,000) and mental disorders (174.0 per 100,000). Suicide rate also increased with age. In 2017, the crude mortality rate of people with disabilities with respiratory diseases was significantly higher than that of other people with disabilities. In contrast, mortality rates were considerably lower among those with intellectual disorders (12.6 per 100,000) and autistic disorder (4.2 per 100,000). According to a study by Giannini et al., the highest rates of suicide were reported among study populations with multiple sclerosis followed by those with spinal cord injury and those with intellectual disability [27]. Mortality due to suicide among those with spinal cord injury decreased in the three injury cohorts; however, it still remained three times higher than that of the general population [28].

Motor vehicle crashes were one of the major causes of ECI-specific mortality between 2008 and 2017. The mortality rate due to motor vehicle crashes among people with disabilities in 2017 (30.8 per 100,000) was lower than that in 2008 (47.2 per 100,000); however, it remained higher than that of the general population. The crude mortality rate due to traffic accidents in Korea in 2007 (9.8 per 100,000) was found to be lower than in the United States (11.4 per 100,000); however, it was significantly higher than that in Japan (3.5 per 100,000) and France (5.3 per 10,000) [29]. According to OECD (Organization for Economic Co-operation and Development) statistics, the mortality rate due to traffic accidents in Korea is steadily decreasing; however, it is still higher than that in other developed countries. The decrease in mortality due to traffic accidents may be attributed to the government’s intense implementation of measures such as traffic safety campaigns, installation of safety facilities (such as drowsy shelters to improve road safety), reinforcement of traffic accident prevention functions, and intensive enforcement of traffic law violations [30]. In 2017, when considering types of disabilities among people with disabilities, those with hearing impairment (44.6 per 100,000) showed the highest mortality rate due to transportation accidents, which may be hypothesized as being a direct result of hearing problems.

Falls were one of most prevalent causes of ECI-specific mortality. From 2008 to 2017, the mortality rate due to falls among people with disabilities was maintained at approximately 20 per 100,000, without major changes in trends during this period. The corresponding mortality rate among the general population was maintained at around 5 per 100,000. In the transition into an industrialized society, it is inevitable to have offices in high-rise buildings, and accordingly, the increase in accidents due to free falls may be interpreted in the same context as the exponential increase in traffic accidents [31].

In this study, the ECI among people with disabilities was higher than that of the general population in South Korea. The ECI types can be classified into unintentional injuries such as motor vehicle crashes, falls, drowning, burns, and poisoning, and intentional injuries such as suicide and homicides [32,33,34]. Intentional injury is related to poverty, job problems, and disease problems, and it is known that people with disabilities experience these problems [35,36,37]. However, categorizing suicide as “intentional” injury is a problem. We believe that suicide can be caused by a combination of intentional and unintentional factors.

There are some limitations in this study. These data dealt with mortality due to ECIs among people with disabilities in 2008–2017. The 2017 data were the most recently available and hence, we were not able to evaluate current or more recent data. The subjects of this study are those with a registered disability in South Korea. The registered people with disabilities in South Korea were grouped based on their disabilities into 15 categories such that the categories were heterogenous. ECI mortality rates differed with each type of disability. Therefore, when establishing a strategy to prevent ECI, a plan for each type of disability should be established. This study was not designed to explore causality, so we cannot explain the reasons behind the higher incidence of accidents in people with disabilities. As a follow-up study, it is necessary to investigate the causal relationships of the occurrence of ECIs in persons with disabilities. We also could not accurately compare people with disabilities and those without disabilities in this study due to the protection of personal information among the latter; hence, aggregated data for the general population was used as a proxy. However, the gap between the general population and people without disabilities was not large because the proportion of people with disabilities in the entire population of Korea is about 5%. Another limitation is that the mortality data used in this study were based on death reports, which may have a lower accuracy than the true mortality rate of the general population. Despite these limitations, this study may have significant implications, as it looked at ECI-specific data among people with disabilities over the course of 10 years and thus is the first study to evaluate mortality due to ECIs among people with disabilities in South Korea.

## 5. Conclusions

The purpose of this study was to analyze the current status of ECI-related deaths among people with disabilities in Korea from 2008 to 2017 and to provide evidence that warrants the establishment of preventable intervention strategies. The death toll among people with disabilities increased during the 10-year period, and the mortality rate due to ECIs out of the total number of deaths ranged from 6.5% to 9.2%. The proportion of ECI-related deaths among those with disabilities was the highest owing to suicides, motor vehicle crashes, and falls. The results in this study provide information that can be accessed by government agencies to develop policies, which by implementing, can reduce the number of ECI deaths among people with disabilities. Therefore, our results suggest that ECIs are public health problems in South Korea, and their prevention needs to be prioritized. ECI surveillance systems and targeted studies are needed for the development of inclusive ECI prevention programs.

## Figures and Tables

**Table 1:** Crude all-cause mortality rates and crude mortality rates due to external causes of injury (ECIs), 2008–2017.

Years	People with Disability			General Population		
	All-Cause Mortality, per 100,000	ECI ^1^-Specific Mortality, per 100,000	ECI/All (%)	All-Cause Mortality, per 100,000	ECI Mortality, per 100,000	ECI/All (%)
2008	2640.8	237.7	9.0	498.2	61.7	12.4
2009	2554.6	241.9	9.5	497.3	65.8	13.2
2010	2614.5	238.2	9.1	512.0	65.5	12.8
2011	2529.4	231.5	9.2	513.6	64.7	12.6
2012	2649.3	218.8	8.3	530.8	61.9	11.7
2013	2560.7	210.6	8.2	526.6	61.3	11.6
2014	2431.3	187.3	7.7	527.3	57.8	11.0
2015	2747.2	196.8	7.2	541.5	56.5	10.4
2016	2813.0	193.5	6.9	549.4	55.2	10.0
2017	2750.6	178.4	6.5	557.3	53.0	9.5

^1^ ECI: external cause of injury.

**Table 2:** Crude mortality rate due to external causes of injury (ECIs) by sex and age of persons with disabilities, 2017 (Unit: per 100,000 people).

Age	Motor Vehicle Crashes	Falls	Drowning	Burns	Poisoning	Suicide	Homicide	Others	Total
**Total**									
0–9	3.8	7.5	0.0	0.0	0.0	0.0	7.5	11.3	30.2
10–19	1.6	4.8	3.2	0.0	1.6	3.2	0.0	7.9	22.2
20–29	6.5	1.1	0.0	0.0	2.2	46.6	2.2	11.9	70.5
30–39	11.0	4.8	1.4	1.4	0.7	49.5	1.4	15.1	86.0
40–49	18.3	6.9	4.5	0.7	1.7	62.9	1.4	25.6	121.9
50–59	22.8	14.0	3.5	1.2	1.2	63.4	2.6	31.3	140.0
60–69	29.7	23.4	2.5	1.4	1.6	54.1	1.3	42.4	156.4
70–79	48.4	28.2	3.7	0.9	2.2	69.1	1.6	73.1	227.2
80+	51.5	49.2	4.7	1.3	4.3	82.5	2.0	177.9	373.4
**Male**									
0–9	5.7	5.7	0.0	0.0	0.0	0.0	5.7	17.2	34.5
10–19	2.4	4.9	2.4	0.0	2.4	2.4	0.0	9.8	24.5
20–29	9.8	1.6	0.0	0.0	1.6	50.9	3.3	13.1	80.4
30–39	15.9	6.0	2.0	2.0	0.0	53.8	0.0	16.9	96.7
40–49	22.1	6.9	5.4	1.0	1.5	71.9	1.5	28.5	138.8
50–59	29.4	18.0	4.9	1.7	1.5	77.7	1.7	40.7	175.8
60–69	39.7	32.7	3.6	2.4	1.5	73.0	0.8	55.5	209.1
70–79	75.7	37.8	4.2	0.8	2.3	105.8	1.9	102.0	330.5
80+	88.8	76.6	9.3	1.9	10.3	159.8	2.8	206.5	556.0
**Female**									
0–9	0.0	11.0	0.0	0.0	0.0	0.0	11.0	0.0	22.0
10–19	0.0	4.5	4.5	0.0	0.0	4.5	0.0	4.5	18.0
20–29	0.0	0.0	0.0	0.0	3.2	38.4	0.0	9.6	51.2
30–39	0.0	2.2	0.0	0.0	2.2	39.9	4.4	13.3	62.1
40–49	9.3	7.0	2.3	0.0	2.3	41.7	1.2	18.5	82.3
50–59	9.1	5.5	0.6	0.0	0.6	33.5	4.3	11.6	65.2
60–69	15.1	9.8	0.9	0.0	1.8	26.2	2.2	23.1	79.1
70–79	23.2	19.4	3.2	1.1	2.1	35.2	1.4	46.5	132.0
80+	31.0	34.1	2.1	1.0	1.0	39.7	1.5	162.1	272.6

**Table 3:** Crude and age-adjusted mortality rates due to external causes of injury (ECIs) by type of disability, 2017 (unit: per 100,000 people).

Disability Type	Motor Vehicle Crashes		Falls		Drowning		Burns		Poisoning		Suicide		Homicide		Others		Total	
	CMR ᵃ	SMR ᵇ	CMR	SMR	CMR	SMR	CMR	SMR	CMR	SMR	CMR	SMR	CMR	SMR	CMR	SMR	CMR	SMR
Physical disability	30.0	12.1	17.1	5.5	2.9	1.4	1.1	0.7	1.5	1.6	57.5	39.0	1.1	0.5	43.5	12.7	154.8	73.5
Disability of brain lesion	34.3	17.9	25.5	7.8	4.4	4.2	1.2	0.3	3.2	0.9	62.2	36.4	3.2	2.0	155.9	87.4	289.9	156.9
Visual impairment	26.5	12.5	24.9	9.7	3.6	1.7	1.2	0.1	1.2	0.6	66.5	41.5	1.6	0.6	38.8	8.8	164.3	75.6
Hearing impairment	44.6	10.1	32.4	5.9	3.8	0.7	1.0	0.5	3.1	0.3	65.9	38.0	1.7	0.5	69.1	12.5	221.8	68.5
Speech disability	25.2	19.2	15.1	4.9	0.0	0.0	0.0	0.0	5.0	1.1	25.2	23.4	0.0	0.0	60.5	21.9	131.1	70.6
Intellectual disability	16.7	20.6	7.1	10.7	3.0	3.1	0.0	0.0	1.0	0.9	12.6	11.3	3.0	4.4	29.3	35.5	72.7	86.4
Autisic disorder	16.8	7.9	16.8	7.3	0.0	0.0	0.0	0.0	0.0	0.0	4.2	2.1	4.2	2.1	16.8	19.0	58.9	38.4
Mental disorder	24.9	13.0	8.0	4.5	7.0	3.6	3.0	2.6	4.0	7.4	174.0	184.8	4.0	4.1	72.6	51.6	297.3	271.7
Kidney dysfunction	43.3	29.9	68.1	48.7	2.5	0.7	0.0	0.0	0.0	0.0	65.6	40.2	0.0	0.0	44.6	34.6	224.0	88.7
Cardiac dysfunction	36.8	5.6	36.8	46.8	0.0	0.0	0.0	0.0	0.0	0.0	0.0	0.0	0.0	0.0	92.1	182.8	165.7	133.4
Respiratory dysfunction	42.6	8.0	42.6	17.7	8.5	1.0	0.0	0.0	0.0	0.0	204.5	40.1	8.5	5.1	68.2	133.6	374.9	205.6
Hepatic dysfunction	8.8	4.2	0.0	0.0	0.0	0.0	0.0	0.0	8.8	13.2	43.8	104.0	0.0	0.0	35.0	70.1	96.3	191.5
Facial dysfunction	0.0	0.0	0.0	0.0	0.0	0.0	0.0	0.0	0.0	0.0	37.2	115.4	0.0	0.0	0.0	0.0	37.2	115.4
Intestinal fistula/urinary fistula	34.4	4.2	27.5	7.2	0.0	0.0	0.0	0.0	0.0	0.0	82.5	16.2	0.0	0.0	34.4	3.0	178.7	30.6
Epilepsy	43.2	19.7	86.4	45.5	0.0	0.0	0.0	0.0	28.8	16.6	115.2	141.4	14.4	7.4	57.6	35.0	345.6	265.7

ᵃ CMR: Crude mortality rate. ᵇ SMR: Standardized mortality rate (age-adjusted mortality).

**Table 4:** Crude mortality rate due to the top three external causes of injury (ECIs) by age (unit: per 100,000 people).

Ages	Suicide			Motor Vehicle Crashes			Falls		
	Respiratory Dysfunction ^1^	Mental Disorder ^2^	Epilepsy ^3^	Hearing Impairment ^1^	Kidney Dysfunction ^2^	Epilepsy ^3^	Epilepsy ^1^	Kidney Dysfunction ^2^	Respiratory Dysfunction ^3^
0–9	0.0	0.0	0.0	0.0	0.0	0.0	0.0	0.0	0.0
10–19	0.0	0.0	0.0	0.0	0.0	0.0	0.0	0.0	0.0
20–29	0.0	525.5	464.0	0.0	0.0	0.0	0.0	0.0	0.0
30–39	0.0	221.2	185.5	0.0	0.0	0.0	0.0	9.6	0.0
40–49	0.0	181.0	0.0	13.8	13.7	0.0	107.7	6.8	0.0
50–59	98.4	179.6	141.0	26.3	8.6	47.0	47.0	14.3	147.6
60–69	175.8	107.6	0.0	26.8	51.0	196.1	294.1	90.6	0.0
70–79	295.5	42.4	505.1	51.8	275.7	0.0	0.0	445.4	26.9
80+	378.4	149.0	0.0	70.6	894.2	0.0	0.0	1490.3	94.6

^1~3^ top 3 rankings.

## Data Availability

Data were collected form publicly archived datasets analyzed or generated during the study and presented in Table 1.
